# Evaluation of the fracture prevention effects of teriparatide and alendronate in patients with frailty: a sub-analysis of the Japanese osteoporosis intervention trial-05

**DOI:** 10.1007/s00774-025-01610-1

**Published:** 2025-05-27

**Authors:** Tatsuya Hosoi, Makoto Yunoki, Shiro Tanaka, Hiroshi Hagino, Satoshi Mori, Satoshi Soen, Sumito Ogawa

**Affiliations:** 1 Department of Geriatric Medicine, Graduate School of Medicine, The University of Tokyo, 7-3-1 Hongo, Bunkyo-Ku, Tokyo 113-8655 Japan; 2 Department of Clinical Biostatistics, Graduate School of Medicine, Kyoto University, Kyoto, Japan; 3 Department of Rehabilitation, Sanin Rosai Hospital, Yonago, Tottori Japan; 4 Seirei Hamamatsu General Hospital, Hamamatsu, Japan; 5 Soen Orthopaedics, Osteoporosis and Rheumatology Clinic, Kobe, Hyogo Japan

**Keywords:** Frailty, Osteoporosis, Fracture, Alendronate, Teriparatide

## Abstract

**Introduction:**

The fracture prevention effects of teriparatide (TPTD) and alendronate (ALN) were evaluated in frail patients using data from the JOINT-05 trial. In addition, predictors of adherence-related treatment discontinuation were evaluated for TPTD and ALN.

**Materials and methods:**

Japanese women aged ≥ 75 years with primary osteoporosis and high fracture risk were randomized to either sequential therapy (TPTD for 72 weeks followed by ALN for 48 weeks) or ALN monotherapy for 120 weeks. Cognitive frailty was defined as an MMSE score ≤ 27, and physical frailty as requiring support or nursing care. Vertebral, non-vertebral, and all fractures were assessed. Adherence-related discontinuations were identified, and baseline predictors were analyzed using multiple regression to calculate odds ratios.

**Results:**

A total of 514 patients with cognitive frailty (254 with TPTD, 260 with ALN) and 204 patients with physical frailty (109 with TPTD, 95 with ALN) were identified. In patients with cognitive frailty, vertebral fracture incidence tended to be lower with TPTD (rate ratio 0.72), but not significantly. In patients with physical frailty, the incidence was significantly lower with TPTD (rate ratio 0.50, *p* < 0.01). Dyslipidemia and serum calcium levels were significant predictors of TPTD discontinuation, whereas cognitive impairment and dyslipidemia were predictors for ALN discontinuation.

**Conclusion:**

In patients with physical frailty, TPTD reduced vertebral fractures significantly more than ALN. However, in cases with cognitive impairment, the results of the JOINT-05 study may not necessarily apply. Assessing the presence of dyslipidemia and cognitive decline may be useful for predicting adherence-related discontinuation.

**Trial registration:**

jRCTs031180235 and UMIN000015573, March 12, 2019.

**Supplementary Information:**

The online version contains supplementary material available at 10.1007/s00774-025-01610-1.

## Introduction

Osteoporosis is a skeletal disorder characterized by low bone mineral density (BMD) and deterioration of bone microarchitecture, leading to increased fracture risk [1, 2]. In Japan, approximately 15.9 million individuals are affected by osteoporosis, and vertebral fractures alone impact around 14.6 million people [3–5]. These fractures not only increase mortality but also impair activities of daily living (ADL) and quality of life (QOL). Although several pharmacological agents are available, more effective treatments are needed to address this growing clinical challenge [2].

Frailty is another critical issue in aging populations, characterized by a decline in physiological reserves and diminished resistance to stressors [6]. Physical frailty, often linked to sarcopenia, is associated with decreased muscle function and an elevated risk of falls and fractures. Cognitive frailty, involving cognitive impairment, is associated with an increased need for care and mortality risk [7]. Notably, physical frailty and cognitive impairment have a bidirectional relationship, as physically frail individuals are at higher risk of developing dementia, while cognitive impairment can accelerate physical decline [8, 9]. Among frail patients, those with osteosarcopenia—the coexistence of sarcopenia and osteoporosis—have a particularly high fracture risk, highlighting the importance of targeted osteoporosis management in this population [10, 11].

Osteoporosis exhibits significant sex-related differences. Estrogen, a critical female hormone, is closely associated with BMD; therefore, women experience a rapid decrease in BMD after menopause [12]. Since men generally attain a higher peak bone mass than women, the prevalence of osteoporosis in women over the age of 50 years is approximately four times higher than that in men of the same age group [13]. Therefore, the JOINT-05 study focused on older Japanese women at high fracture risk and demonstrated that the incidence rate of morphometric vertebral fractures was lower with treatment using once-weekly teriparatide (TPTD) followed by alendronate (ALN), compared with treatment with ALN alone throughout the study [14–16]. In this study, the focus was on patients with frailty, and comprehensive subgroup analyses were conducted using data from JOINT-05. In addition, cases of discontinuation due to poor adherence were identified with the aim of clarifying the baseline predictors associated with discontinuation.

## Materials and methods

### Study design and participants

**Fig. 1:**
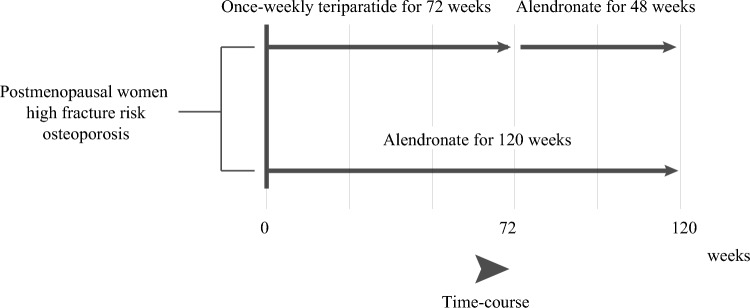
Drug administration periods and study flow by group

The JOINT-05 study was a prospective, randomized, open-label, blinded-endpoint trial conducted between October 2014 and June 2020 at 113 institutions in Japan. This study is a sub-analysis of the JOINT-05 trial, which was conducted as part of the Adequate Treatment of Osteoporosis (A-TOP) research group, and Japanese women aged ≥ 75 years were enrolled if they had primary osteoporosis and were at high risk of fracture. The detailed design, eligibility, and baseline characteristics of JOINT-05 have been described previously [14–16]. Participants were randomly allocated in a 1:1 ratio to two groups: sequential therapy with once-weekly TPTD for 72 weeks followed by once-weekly ALN for 48 weeks (TPTD group); or monotherapy with ALN for 120 weeks (ALN group). Both groups received daily supplements of 400 IU of native vitamin D throughout the entire treatment period. The design diagram is shown in Fig. 1. This study was conducted in accordance with the Declaration of Helsinki and was approved by the central and onsite institutional review boards. Informed consent was obtained from all participants.

This study was a subgroup analysis of the primary and secondary fracture endpoints, specifically aimed at elucidating outcomes in patients with frailty [17]. Physical frailty was defined as a condition requiring support or nursing care, and cognitive frailty was defined as a Mini-Mental State Examination (MMSE) score of 27 or lower [18]. In the two groups randomly assigned in JOINT-5, patients with frailty were identified, and subgroup analyses were performed. In addition, the number of dropouts due to medication non-adherence was identified from the full analysis set. The dataset for these analyses comprised data finalized by the Joint Center for Researchers, Associates and Clinician (JCRAC) Data Center of the National Center for Global Health and Medicine on February 4, 2020, as well as the Week 120 fracture assessment results from the fracture assessment committee in October 2020, and the list of discontinued cases verified during a case review meeting in December 2022.

### Baseline characteristics and outcomes

The baseline characteristics, laboratory measurements, QOL, and dietary intakes were collected at baseline. Baseline patient characteristics included age, age at menopause, years since menopause, body weight, height, and body mass index (BMI; calculated as weight in kilograms divided by height in meters squared). The number and grades of prevalent vertebral fractures, history of prior femoral fractures, osteoporosis treatment, and bisphosphonate use were also recorded. Bone mineral density (BMD, T-score) was measured at the lumbar spine, total hip, and femoral neck using dual-energy X-ray absorptiometry (DXA) at each participating institution. Comorbidities, including hypertension, diabetes mellitus, dyslipidemia, rheumatoid arthritis, osteoarthritis, and other conditions, as well as systolic and diastolic blood pressures, were documented. Cognitive function was evaluated using MMSE scores, categorized as ≥ 28, 24–27, and ≤ 23. Physical function was assessed with the timed up-and-go test and the one-leg standing test with eyes open. Social support levels were classified into five categories: support-required and nursing care levels 1, 2, 3, and 4–5. Laboratory measurements included bone turnover markers [osteocalcin (ng/mL), procollagen type I amino-terminal propeptide (P1NP; μg/L), and tartrate-resistant acid phosphatase-5b (TRACP-5b; mU/dL)], 25-hydroxyvitamin D (25OHVD; ng/mL), pentosidine (pmol/mL), HbA1c (%), total cholesterol (mg/dL), high-density lipoprotein (HDL) cholesterol (mg/dL), low-density lipoprotein (LDL) cholesterol (mg/dL), triglycerides (mg/dL), estimated glomerular filtration rate (eGFR; mL/min/1.73 m^2^), creatinine (mg/dL), albumin (g/dL), and calcium (mg/dL). Back pain severity was assessed using a visual analog scale (VAS), ranging from 0 to 100 points, and QOL was measured using the EQ-5D questionnaire. Nutrient intake was evaluated through a Food Frequency Questionnaire [19].

The study’s primary endpoint was morphometric vertebral fractures, and the secondary endpoints included clinical vertebral fractures, progression of vertebral fractures, non-vertebral fractures, and the overall incidence of all fractures at 0, 24, 48, 72, and 120 weeks. Adherence-related treatment discontinuation was defined based on pre-specified trial criteria, including voluntary withdrawal, prolonged interruption of TPTD therapy (> 8 weeks), complete discontinuation of ALN therapy, or other investigator-determined reasons. Discontinuation was determined using both physician judgment and patient self-reporting and was recorded at the last visit where these criteria were met. In the analyses of patient background characteristics related to adherence-related treatment discontinuation, compliance-related treatment discontinuation was defined as the primary outcome, and predictors were identified using univariable logistic regression analysis.

### Statistical analysis

Numerical and categorical data are described by means and standard deviation (SD) values and proportions, respectively. In the fracture analyses, multivariable Poisson regression models were used to estimate the rate ratios between the TPTD group and the ALN group, along with their 95% confidence intervals (CIs), using the generalized estimating equation (GEE) approach. The GEE-Poisson regression included age, counts, and maximum grades of prevalent vertebral fractures, history of proximal femoral fractures, and BMD at baseline as covariates and individual and institute as clusters. These variables were selected based on their specification in the original JOINT-05 study protocol and their established clinical relevance to fracture risk [14]. Superiority hypotheses for the incidences of morphometric vertebral fractures, any fractures, clinical vertebral fractures, and the progression of vertebral fractures and the non-inferiority hypothesis for non-vertebral fractures, defined as the upper limit of the 95% CI for the rate ratio being less than 1.96, were tested [15]. In the analysis of the contributions of patient background characteristics to adherence-related discontinuation, logistic regression models were used to calculate odds ratios (ORs) and 95% CIs. Potential predictors of adherence-related discontinuation were initially screened by univariable regression with *P *value < 0.05, followed by fitting with multivariable regression. Missing covariates were handled by multiple imputation, applying a monotone model specification. Multiplicity adjustment was not performed, because this was an explanatory analysis, and *p* values < 0.05 were considered significant. All analyses were performed using SAS software version 9.4 (SAS Institute, Cary, NC).

## Results

### Patients

In the JOINT-05 study, a total of 985 cases were initially selected [15, 16]. For this analysis, 16 cases with missing MMSE data and 1 case with missing information on nursing support status were excluded. Consequently, the final analysis included 514 cases with cognitive frailty (455 cases with MMSE scores above 27) and 204 participants with physical frailty (780 cases categorized as ‘Nursing not required’). Of the patients with cognitive frailty, 254 participants were allocated to the TPTD group and 260 were allocated to the ALN group. Of the patients with physical frailty, 109 were assigned to the TPTD group and 95 were assigned to the ALN group.

The baseline characteristics of both groups are shown in Table 1 and Supplementary Tables 1 to 3. As shown in Table 1, the baseline characteristics were well balanced between the treatment groups for both patients with cognitive frailty and those with physical frailty. For patients with cognitive frailty, the mean age (SD) was 82.3 (4.7) years in the TPTD group and 82.4 (4.9) years in the ALN group; for those with physical frailty, it was 83.9 (5.0) years in the TPTD group and 84.1 (5.1) years in the ALN group. The mean BMD T-score values ranged from − 3.1 to − 3.4, with no significant differences observed between treatment groups, and 68–78% of participants had prevalent vertebral fractures. Of the patients with cognitive frailty, approximately 40% had a maximum fracture grade of 3, whereas this proportion was around 50–55% in patients with physical frailty. The physical frailty group exhibited a tendency toward a higher incidence and greater severity of pre-existing fractures, suggesting that these prior fractures may have contributed to declines in physical function.

**Table 1:** Baseline characteristics of postmenopausal women with severe osteoporosis and frailty

Characteristic	Cognitive frailty						Physical frailty						
	Teriparatide (*N* = 254)			Alendronate (*N* = 260)			Teriparatide (*N* = 109)				Alendronate (*N* = 95)		
	Mean	SD	Missing	Mean	SD	Missing	Mean	SD	Missing	Mean		SD	Missing
Age (y)	82.3	4.7	0	82.4	4.9	0	83.9	5.0	0	84.1		5.1	0
Age at menopause (y)	49.5	4	0	49.1	4.4	0	49.1	4.3	0	49.3		4.4	0
Years from menopause	32.9	6.4	0	33.4	6.8	0	34.8	6.9	0	34.8		6.9	0
Height (cm)	145.5	6.7	0	145	6.3	0	145	7.3	0	144.6		6	0
Weight (kg)	46.6	8.4	0	46.3	8.1	0	48.2	10.7	0	46.8		8.8	0
BMI (kg/m^2^)	22	3.6	0	22	3.6	0	22.9	4.5	0	22.4		4	0
< 18.5 kg/m^2^	14.2%		0	15.4%		0	16.5%		0	21.1%			0
18.5 to 24.9 kg/m^2^	66.5%		0	65.4%		0	52.3%		0	56.8%			0
≥ 25 kg/m^2^	19.3%		0	19.2%		0	31.2%		0	22.1%			0
Count of prevalent vertebral fractures	1.7	1.8	0	1.8	2	0	1.9	2.1	0	2.2		2.2	0
0	32.3%		0	30.8%		0	24.8%		0	22.1%			0
1	24.0%		0	26.5%		0	29.4%		0	25.3%			0
2	17.3%		0	13.1%		0	20.2%		0	16.8%			0
3	11.4%		0	11.5%		0	9.2%		0	18.9%			0
4	4.7%		0	6.2%		0	5.5%		0	4.2%			0
5 or more	10.2%		0	11.9%		0	11.0%		0	12.6%			0
Grade 1	8.7%		0	9.6%		0	8.3%		0	8.4%			0
Grade 2	16.1%		0	16.9%		0	13.8%		0	14.7%			0
Grade 3	42.9%		0	42.7%		0	53.2%		0	54.7%			0
History of proximal femoral fractures	17.7%		0	16.5%		0	21.1%		0	29.5%			0
Prior treatment	52.0%		0	55.8%		0	53.2%		0	63.2%			0
Prior bisphosphonates	28.3%		0	30.5%		1	26.6%		0	30.9%			1
BMD (T-score)	− 3.2	1.9	25	− 3.4	1.8	29	− 3.1	1.8	13	− 3.3		2	11
BMD at L2-L4 (T-score)	− 2.2	1.6	129	− 2.5	1.4	127	− 2.3	1.7	54	− 2.2		1.5	52
Hypertension	41.7%		0	41.2%		0	41.3%		0	37.9%			0
Diabetes	9.8%		0	11.2%		0	7.3%		0	11.6%			0
Dyslipidemia	15.4%		0	18.8%		0	14.7%		0	15.8%			0
Rheumatoid arthritis	0.4%		0	1.5%		0	1.8%		0	2.1%			0
Osteoarthritis	0.0%		0	0.4%		0	0.0%		0	0.0%			0
Others	31.9%		0	35.4%		0	33.9%		0	31.6%			0
Systolic blood pressure (mmHg)	133.5	19.7	39	135.3	20.3	44	132.9	17.9	14	138		18.2	11
Diastolic blood pressure (mmHg)	73.5	13	39	73.3	13.1	44	73.3	12	14	75.5		11.6	11
MMSE	23.7	4.3	0	24.1	4.1	0	24.5	5.6	1	24.3		6.2	0
28 or more	0.0%		0	0.0%		0	32.4%		1	32.6%			0
24 to 27	74.0%		0	72.7%		0	39.8%		1	35.8%			0
23 or less	26.0%		0	27.3%		0	27.8%		1	31.6%			0
Timed up-and-go test (second)	14.8	9.6	4	15	12.6	3	18.6	11.4	1	18.2		13.1	1
One leg standing (second)	10.9	17.6	5	10.4	16.7	4	7.4	15.2	1	5.9		13.3	1
Nursing level (support-required)	13.4%		0	10.8%		0	56.9%		0	52.6%			0
Nursing level (level 1)	7.9%		0	6.2%		0	21.1%		0	18.9%			0
Nursing level (level 2)	3.9%		0	4.6%		0	12.8%		0	17.9%			0
Nursing level (level 3)	2.8%		0	1.5%		0	7.3%		0	4.2%			0
Nursing level (level 4 or 5)	0.8%		0	1.5%		0	1.8%		0	6.3%			0

In patients with cognitive frailty, the distribution of MMSE scores was similar between treatment groups, with approximately 73% of participants scoring between 24 and 27, and 27% scoring 23 or below. In patients with physical frailty, more than half of the participants were at the support-required level, whereas those requiring intensive nursing care accounted for less than 10%. The relationship between physical function and cognitive function was summarized as follows: of patients with cognitive frailty, approximately 25–30% required nursing care. In contrast, approximately 67% of patients with physical frailty had MMSE scores of 27 or less. Overall, longitudinal changes in MMSE scores at 120 weeks were minimal for both cognitive and physical frailty patients (Supplementary Table 4).

### Morphometric vertebral fractures

In the fracture risk analysis (Table 2), the incidence of morphometric vertebral fractures in patients with cognitive frailty was lower in the TPTD group; however, there was no significant difference between the TPTD group (41 fractures per 344.7 person-years; annual incidence rate of 0.1190) and the ALN group (70 fractures per 422.4 person-years; annual incidence rate of 0.1657), with a rate ratio of 0.72 (95% CI: 0.38–1.34, *P* = 0.30). In contrast, in patients with physical frailty, the incidence of morphometric vertebral fractures was significantly lower in the TPTD group (14 fractures per 150.6 person-years; annual incidence rate of 0.0929) than in the ALN group (31 fractures per 157.9 person-years; annual incidence rate of 0.1963), with a rate ratio of 0.50 (95% CI: 0.37–0.68, *P* < 0.01). These results reflect the full observation period of 0–120 weeks. In a post hoc analysis of the period from 72 to 120 weeks, a consistent trend was observed. In patients with cognitive frailty, the rate ratio was 0.38 (95% CI: 0.13–1.12, *P* = 0.079), indicating no statistically significant difference. In patients with physical frailty, the rate ratio was 0.21 (95% CI: 0.05–0.96, *P* = 0.044), showing a significant reduction in fracture risk with sequential therapy, consistent with the overall findings.

**Table 2:** Fracture incidence by treatment group and frailty

	Teriparatide to alendronate				Alendronate to alendronate							
	Count	Person	Person-years	Annual incidence rate	Count	Person	Person-years	Annual incidence rate	Rate ratio*	95% CI		*p*
Cognitive Frailty												
Morphometric vertebral fracture	41	30	344.7	0.119	70	51	422.4	0.1657	0.72	0.38	1.34	0.3
Secondary endpoint												
All fractures	50	40	370.5	0.135	77	60	455.4	0.1691	0.8	0.53	1.21	0.29
Clinical vertebral fracture	2	2	344.7	0.0058	3	2	422.4	0.0071	0.84	0.15	4.82	0.85
Progression of vertebral fracture	18	17	344.7	0.0522	24	21	422.4	0.0568	0.97	0.42	2.23	0.95
Non-vertebral fracture	12	8	370.5	0.0324	13	13	455.4	0.0285	Not estimable			
Secondary endpoint (fractures at specific skeletal sites)												
Forearm	2	2	370.5	0.0054	3	3	455.4	0.0066				
Humerus	1	1	370.5	0.0027	0	0	455.4	0				
Femur	2	2	370.5	0.0054	4	4	455.4	0.0088				
Lower leg	0	0	370.5	0	0	0	455.4	0				
Clavicle	0	0	370.5	0	0	0	455.4	0				
Pelvis	0	0	370.5	0	2	2	455.4	0.0044				
Rib	3	1	370.5	0.0081	1	1	455.4	0.0022				
Other	4	4	370.5	0.0108	3	3	455.4	0.0066				
Physical Frailty												
Morphometric vertebral fracture	14	10	150.6	0.0929	31	21	157.9	0.1963	0.5	0.37	0.68	< 0.01
Secondary endpoint												
All fractures	24	16	160.6	0.1495	33	25	167.8	0.1967	0.75	0.39	1.45	0.39
Clinical vertebral fracture	3	2	150.6	0.0199	4	3	157.9	0.0253	0.82	0.17	3.86	0.8
Progression of vertebral fracture	3	3	150.6	0.0199	9	8	157.9	0.057	Not estimable			
Non-vertebral fracture	10	6	160.6	0.0623	5	5	167.8	0.0298	2.79	1.07	7.26	0.04
Secondary endpoints (fractures at specific skeletal sites)												
Forearm	1	1	160.6	0.0062	0	0	167.8	0				
Humerus	0	0	160.6	0	0	0	167.8	0				
Femur	1	1	160.6	0.0062	3	3	167.8	0.0179				
Lower leg	0	0	160.6	0	0	0	167.8	0				
Clavicle	0	0	160.6	0	0	0	167.8	0				
Pelvis	1	1	160.6	0.0062	0	0	167.8	0				
Rib	4	2	160.6	0.0249	1	1	167.8	0.006				
Other	3	3	160.6	0.0187	1	1	167.8	0.006				

*GEE−Poisson regression adjusted for treatment, age, counts and maximum grade of prevalent vertebral fractures, history of proximal femur fractures, and BMD as covariates and individual and institute as cluster

For the secondary endpoints, a higher fracture rate was observed in the TPTD group for non-vertebral fractures (non-inferiority test, *P* = 0.04), although the superiority test showed no significant difference between the two groups. Analyses of the other secondary endpoints showed no significant treatment effects.

### Adherence-related treatment discontinuation

During the observation period, adherence-related discontinuation was observed in 30.4% (156/514) of patients with cognitive frailty and 28.4% (58/204) of patients with physical frailty (Supplementary Table 5). In the ALN group, the rate of adherence-related discontinuation was significantly higher in patients with cognitive frailty than in those without (31.5% vs. 20.7%, *p* < 0.01). No other significant differences in discontinuation rates were observed based on the presence or absence of cognitive or physical frailty.

Table 3 shows the predictors of discontinuation based on baseline measurements. In the TPTD group, dyslipidemia and calcium levels were significantly associated with adherence-related discontinuation (odds ratio [OR]: 0.53, 95% CI: 0.29–0.99, *p* = 0.05; OR: 0.54, 95% CI: 0.30–0.96, *p* = 0.03, respectively). In the ALN group, MMSE scores and dyslipidemia were predictors of discontinuation (OR: 0.90, 95% CI: 0.85–0.95, *p* < 0.01; OR: 0.38, 95% CI: 0.19–0.77, *p* = 0.01, respectively). When analyzing both groups together, MMSE scores, the number of prevalent vertebral fractures, dyslipidemia, weight, P1NP, and the number of teeth extracted in the previous year were significantly associated with adherence-related discontinuation. The ORs (95% CI) for adherence-related discontinuation and all patient background factors examined in this study are shown in Supplementary Table 6.

**Table 3:** Multivariable logistic regression analysis of associations between adherence-related treatment discontinuation and significant parameters on univariable analysis

Treatment	Predictor	Odds ratio	95% CI		*p*
All	MMSE (1-point increase)	0.95	0.92	0.99	0.01
	Count of prevalent vertebral fractures (1-count increase)	0.91	0.84	0.99	0.03
	Dyslipidemia	0.5	0.32	0.79	< 0.01
	Weight (1-kg increase)	0.98	0.96	1	0.03
	P1NP (1-μg/L increase)	1.01	1	1.01	0.01
	Number of teeth extracted last year	0.91	0.84	0.98	0.01
TPTD	Dyslipidemia	0.53	0.29	0.99	0.05
	Ca (1-mg/dL increase)	0.54	0.3	0.96	0.03
ALN	MMSE (1-point increase)	0.9	0.85	0.95	< 0.01
	Dyslipidemia	0.38	0.19	0.77	0.01

^*^Predictors were selected using univariable logistic regression

## Discussion

In the JOINT-05 trial, older and frail adults, who are often excluded from clinical studies, were included in the study population. This research specifically focused on these patient groups in its subgroup analyses. Therefore, the efficacy and safety of TPTD and ALN in older and frail adults were evaluated in greater detail, providing valuable insights that may inform treatment strategies in clinical practice. This subgroup analysis provided modest evidence of differences in treatment efficacy, adherence rates, and unique challenges faced by frail patients.

Frailty and osteoporosis share numerous common factors associated with aging, including decreases in sex and anabolic hormones, vitamin D deficiency, and decreased mechanical loading [20, 21]. These shared factors contribute to a close relationship between frailty and osteoporosis, making their co-occurrence common. The coexistence of osteoporosis and frailty is associated with decreased skeletal muscle mass and strength, increasing the risk of falls. A bidirectional relationship between frailty and falls has also been suggested, since falls can lead to a fear of falling, reducing activity levels, and thereby contributing to the onset and progression of frailty [22, 23]. In addition, in community-dwelling older adults, cognitive frailty has a significant impact on functional status, the onset of dementia, and the occurrence of falls. As a result, exploring and identifying effective osteoporosis treatments for frail patients is essential [24].

The present analysis demonstrated that TPTD significantly reduced the incidence of vertebral fractures compared with ALN in patients with physical frailty. The effectiveness of TPTD observed in our study aligns with the previous findings, further supporting its role in fracture prevention [15, 16]. Moreover, our findings highlight that TPTD is effective even in patients with frailty, a population that is often excluded from clinical trials despite its growing prevalence in aging societies. Notably, the incidence of morphometric vertebral fractures in the monotherapy group among physically frail patients was 1.3 times higher than the incidence observed in the overall JOINT-05 results. On the other hand, in the sequential therapy group, the incidence of morphometric vertebral fractures was comparable to that in the JOINT-05 results [16]. These findings suggest that physically frail patients represent a high-risk population for fractures, likely due to factors such as impaired muscle strength, balance deficits, and poor bone quality. Given this elevated risk, the use of bone-forming agents should be actively considered to optimize fracture prevention in this vulnerable population.

Conversely, the results of the JOINT-05 trial might not fully apply to patients with cognitive frailty. However, in the TPTD group, the rate ratio of 0.72, which was not significant, was comparable to the rate ratio of 0.78 observed in the JOINT-05 trial. This study and the previous one reported that cognitive impairment in the ALN group was associated with adherence-related treatment discontinuation [25]. Therefore, it was hypothesized that ALN treatment discontinuation would increase, giving TPTD a potential advantage for patients with cognitive frailty; however, the results were contrary to this expectation. Although a limited sample size could have contributed to this outcome, the underlying causes remain unclear. Further research is needed to determine whether specific factors, such as the use of other medications such as anti-dementia drugs, may modulate the effects of TPTD in patients with cognitive frailty [11].

Medication adherence is crucial in osteoporosis treatment, with a strong positive association between adherence rates and fracture risk reduction [26]. However, adherence tends to be lower in older adults, highlighting the need for appropriate medication guidance [27]. The relationships between participants’ baseline clinical parameters and discontinuation were partially discussed in a previous sub-analysis of the JOINT-05 trial, and consistent results were generally shown in the present-study population [25]. It is known that improving medication adherence requires patients to recognize the importance of lifestyle modifications and to actively engage in self-care behaviors. This aligns with our finding that higher MMSE scores were associated with better adherence, likely because cognitive function plays a crucial role in understanding the necessity and benefits of treatment. Nevertheless, the mechanisms underlying the association between dyslipidemia and improved adherence remain unclear. One possible explanation is that patients with dyslipidemia may already have established medication-taking habits due to long-term use of lipid-lowering agents, which could contribute to better adherence to osteoporosis treatment. Additionally, these patients may visit hospitals more frequently for blood test monitoring, providing more opportunities for adherence reinforcement. Interestingly, the present study identified a new potential association between the ‘number of teeth extracted last year’ and medication adherence. Patients undergoing dental treatments might have heightened awareness of osteoporosis treatment-related adverse events, such as bisphosphonate-related osteonecrosis of the jaw (BRONJ), prompting patients to reassess their medication adherence.

The present study has several limitations. First, although the patients’ background characteristics were well balanced between the treatment groups, this study was based on data from the JOINT-05 trial and was not a strictly randomized trial. Second, the sample size was not large enough to detect differences between the treatment groups in patients with frailty. However, the direction of the results from the JOINT-05 trial was consistent in patients with frailty, suggesting the robustness of the findings and the potential utility of TPTD in older adults. Furthermore, as is generally the case for studies derived from the JOINT-05 trial, the eligibility criteria for this study were restricted to osteoporosis patients with a high fracture risk, excluding those in the low-risk group. Given the wide range of fracture risk in patients eligible for bisphosphonate treatment, additional studies may be needed to evaluate outcomes in patients with lower fracture risk. In addition to fracture risk stratification, mobility is another critical factor influencing treatment efficacy. While teriparatide has been shown to improve both bone mass and quality, its effects may be influenced by patients' mobility levels. Since mechanical loading plays a role in bone remodeling, severely frail patients with limited mobility might experience reduced benefits. However, the previous studies have shown that teriparatide enhances bone material properties independently of mechanical stress, suggesting that it may still provide fracture prevention benefits even in populations with restricted mobility [28].

Despite these limitations, the present study demonstrated that TPTD reduced the incidence of vertebral fractures significantly more than ALN in patients with physical frailty. However, in cases of cognitive decline, the applicability of the JOINT-05 study findings may be limited. The present findings suggest that assessing frailty in older adults could play a crucial role in managing osteoporosis and making informed decisions about fracture prevention.

## Supplementary Information

Below is the link to the electronic supplementary material.

Supplementary file1 (DOCX 108 KB)
