# Supplementary material for: Evaluation of the fracture prevention effects of teriparatide and alendronate in patients with frailty: a sub-analysis of the Japanese osteoporosis intervention trial-05
Source: J Bone Miner Metab. 2025 May 27;43(4):448–57. doi: 10.1007/s00774-025-01610-1 (PMC12279556; doi:10.1007/s00774-025-01610-1)
Supplement: Supplementary file 1 — Supplementary file1 (DOCX 108 KB) [file 774_2025_1610_MOESM1_ESM.docx]

**Article title:** Evaluation of the Fracture Prevention Effects of Teriparatide and Alendronate in Patients with Frailty: A Sub-Analysis of the Japanese Osteoporosis Intervention Trial-05

**Journal name:** *Journal of Bone and Mineral*

**Authors:** Tatsuya Hosoi^1^, Makoto Yunoki^1^, Shiro Tanaka^2^, Hiroshi Hagino^3^, Satoshi Mori^4^, Satoshi Soen^5^, Sumito Ogawa^1^

**Affiliations:**

^1^ Department of Geriatric Medicine, Graduate School of Medicine, The University of Tokyo, Tokyo, Japan

^2^ Department of Clinical Biostatistics, Graduate School of Medicine, Kyoto University, Kyoto, Japan

^3^ Department of Rehabilitation, Sanin Rosai Hospital, Yonago, Tottori, Japan

^4^ Seirei Hamamatsu General Hospital, Hamamatsu, Japan

^5^ Soen Orthopaedics, Osteoporosis and Rheumatology Clinic, Kobe, Hyogo, Japan

**Corresponding author:** Sumito Ogawa

Department of Geriatric Medicine, Graduate School of Medicine, The University of Tokyo

7-3-1 Hongo, Bunkyo-ku, Tokyo 113-8655, Japan

Email: suogawa@m.u-tokyo.ac.jp

**Supplementary Table 1. Laboratory measurements in postmenopausal women with severe osteoporosis and frailty**

|  | **Cognitive Frailty** | | | | | |  | **Physical Frailty** | | | | | | |
| --- | --- | --- | --- | --- | --- | --- | --- | --- | --- | --- | --- | --- | --- | --- |
|  | **Teriparatide (N=254)** | | | **Alendronate (N=260)** | | |  | **Teriparatide (N=109)** | | | | **Alendronate (N=95)** | | |
|  | Mean | SD | Missing | Mean | SD | Missing |  | Mean | SD | Missing | Mean | | SD | Missing |
| Osteocalcin (ng/mL) | 19.8 | 12.9 | 4 | 19.6 | 12.6 | 0 |  | 20 | 14.2 | 1 | 20.1 | | 14.6 | 0 |
| P1NP (μg/L) | 63.3 | 45.9 | 3 | 57.8 | 49.7 | 0 |  | 71.2 | 56.4 | 1 | 57.4 | | 37 | 0 |
| TRACP-5b (mU/dL) | 510.7 | 236.8 | 4 | 488.3 | 227.1 | 0 |  | 509.9 | 236.5 | 1 | 491.1 | | 233.1 | 0 |
| 25OHVD (ng/mL) | 16.7 | 5.7 | 4 | 17.3 | 5.6 | 0 |  | 16.6 | 6.4 | 2 | 17.1 | | 5.9 | 0 |
| Pentosidine (pmol/mL) | 37.3 | 31.8 | 8 | 35.8 | 30.7 | 3 |  | 41.4 | 36.4 | 3 | 39.5 | | 29.1 | 1 |
| Corrected pentosidine (pmol/mL) | 49.2 | 28.8 | 8 | 46.4 | 19.8 | 3 |  | 52.7 | 36 | 4 | 49.8 | | 20.8 | 1 |
| HbA1C (%) | 5.9 | 0.6 | 3 | 5.9 | 0.8 | 0 |  | 6 | 0.6 | 1 | 6 | | 0.7 | 0 |
| Total cholesterol (mg/dL) | 199 | 35.7 | 3 | 197.7 | 38.7 | 0 |  | 197.9 | 38 | 1 | 192.8 | | 35 | 0 |
| HDL cholesterol (mg/dL) | 61.5 | 16.5 | 3 | 58.9 | 14.9 | 0 |  | 60.9 | 14.6 | 1 | 58.8 | | 14.3 | 0 |
| LDL cholesterol (mg/dL) | 112.2 | 30.8 | 3 | 113.1 | 32.5 | 0 |  | 111.6 | 31.8 | 1 | 107.5 | | 27.8 | 0 |
| Triglyceride (mg/dL) | 111.9 | 50.6 | 3 | 113 | 54.9 | 0 |  | 114.9 | 47.5 | 1 | 117.8 | | 52.3 | 0 |
| eGFR (mL/min/1.73m^2^) | 62.6 | 19.3 | 3 | 60.4 | 17 | 0 |  | 59.5 | 19 | 1 | 61.7 | | 21.6 | 0 |
| Creatinine (mg/dL) | 0.7 | 0.3 | 3 | 0.7 | 0.2 | 0 |  | 0.8 | 0.3 | 1 | 0.7 | | 0.3 | 0 |
| Urine creatinine (mg/dL) | 78.7 | 60.9 | 8 | 75.1 | 50.6 | 2 |  | 79.8 | 58.9 | 4 | 78.2 | | 47.1 | 1 |
| Albumin (g/dL) | 4.1 | 0.4 | 3 | 4.1 | 0.4 | 0 |  | 4 | 0.4 | 1 | 4.1 | | 0.4 | 0 |
| Ca (mg/dL) | 9.5 | 0.6 | 3 | 9.5 | 0.5 | 0 |  | 9.5 | 0.6 | 1 | 9.5 | | 0.5 | 0 |
| Urine Ca (mg/dL) | 10.6 | 8.8 | 8 | 11 | 8.2 | 2 |  | 10.3 | 8.1 | 4 | 11 | | 8.2 | 1 |

**Supplementary Table 2. Visual Analog Scale and EQ-5D Questionnaire Scores of postmenopausal women with severe osteoporosis and frailty**

|  | **Cognitive Frailty** | | | | | |  | **Physical Frailty** | | | | | | |
| --- | --- | --- | --- | --- | --- | --- | --- | --- | --- | --- | --- | --- | --- | --- |
|  | **Teriparatide (N=254)** | | | **Alendronate (N=260)** | | |  | **Teriparatide (N=109)** | | | | **Alendronate (N=95)** | | |
|  | Mean | SD | Missing | Mean | SD | Missing |  | Mean | SD | Missing | Mean | | SD | Missing |
| VAS at rest (0 to 100 points) | 21.1 | 25.3 | 1 | 25.2 | 27.6 | 0 |  | 23.1 | 27.4 | 1 | 28.5 | | 27.2 | 0 |
| VAS on motion (0 to 100 points) | 35.8 | 30.6 | 1 | 36.3 | 31.3 | 0 |  | 37.9 | 30.4 | 1 | 42.2 | | 31.3 | 0 |
| EQ-5D (mobility) | 1.6 | 0.5 | 1 | 1.6 | 0.5 | 0 |  | 1.8 | 0.5 | 1 | 1.7 | | 0.5 | 0 |
| EQ-5D (self-care) | 1.3 | 0.5 | 1 | 1.3 | 0.5 | 0 |  | 1.5 | 0.6 | 1 | 1.5 | | 0.5 | 0 |
| EQ-5D (usual activities) | 1.6 | 0.5 | 1 | 1.5 | 0.6 | 0 |  | 1.8 | 0.5 | 1 | 1.6 | | 0.5 | 0 |
| EQ-5D (pain/discomfort) | 1.8 | 0.5 | 1 | 1.8 | 0.6 | 0 |  | 1.9 | 0.6 | 1 | 1.9 | | 0.5 | 0 |
| EQ-5D (anxiety/depression) | 1.4 | 0.5 | 1 | 1.3 | 0.5 | 0 |  | 1.5 | 0.5 | 1 | 1.4 | | 0.6 | 0 |
| JOQOL (item 1, pain) | 3.2 | 1.6 | 1 | 3.2 | 1.7 | 0 |  | 3.4 | 1.6 | 1 | 3.4 | | 1.6 | 0 |
| JOQOL (item 2, pain) | 2.2 | 1.3 | 1 | 2.4 | 1.4 | 0 |  | 2.5 | 1.4 | 1 | 2.5 | | 1.4 | 0 |
| JOQOL (item 3, ADL) | 2.7 | 1.7 | 1 | 2.5 | 1.7 | 0 |  | 3.5 | 1.6 | 1 | 3.6 | | 1.7 | 0 |
| JOQOL (item 4, ADL) | 2.7 | 1.3 | 1 | 2.8 | 1.4 | 0 |  | 3.3 | 1.3 | 1 | 3.3 | | 1.2 | 0 |
| JOQOL (item 5, fall/anxiety) | 2.9 | 1.3 | 1 | 2.8 | 1.4 | 0 |  | 2.6 | 1.3 | 1 | 2.5 | | 1.3 | 0 |
| JOQOL (item 6, fall/anxiety) | 3.4 | 1.3 | 1 | 3.3 | 1.4 | 0 |  | 2.9 | 1.2 | 1 | 3 | | 1.3 | 0 |

**Supplementary Table 3. Nutrient intakes in postmenopausal women with severe osteoporosis and frailty**

|  | **Cognitive Frailty** | | | | | |  | **Physical Frailty** | | | | | | |
| --- | --- | --- | --- | --- | --- | --- | --- | --- | --- | --- | --- | --- | --- | --- |
|  | **Teriparatide (N=254)** | | | **Alendronate (N=260)** | | |  | **Teriparatide (N=109)** | | | | **Alendronate (N=95)** | | |
|  | Mean | SD | Missing | Mean | SD | Missing |  | Mean | SD | Missing | Mean | | SD | Missing |
| Alcohol intake (g/day) | 6.8 | 31 | 11 | 8.6 | 36.6 | 6 |  | 4.9 | 22.2 | 3 | 6.8 | | 33 | 5 |
| Energy intake (kcal/day) | 1526.5 | 339.9 | 11 | 1509.6 | 332.7 | 6 |  | 1499.7 | 323.5 | 3 | 1500.3 | | 317.9 | 5 |
| Protein intake (g/day) | 71.3 | 21.5 | 11 | 70.1 | 20.1 | 6 |  | 70.1 | 21.2 | 3 | 69.2 | | 21.5 | 5 |
| Fat intake (g/day) | 55.5 | 15.3 | 11 | 54.1 | 16.3 | 6 |  | 55.6 | 14.8 | 3 | 53.9 | | 16 | 5 |
| Carbohydrate intake (g/day) | 187.6 | 44.7 | 11 | 187.3 | 44.9 | 6 |  | 182.4 | 43.8 | 3 | 186.7 | | 41.8 | 5 |
| Salt intake (g/day) | 10 | 1.9 | 11 | 10.1 | 1.9 | 6 |  | 9.7 | 1.8 | 3 | 10 | | 1.9 | 5 |
| Ca intake (mg/day) | 486.6 | 161.8 | 11 | 490.3 | 169.5 | 6 |  | 482 | 145.1 | 3 | 475.5 | | 167.3 | 5 |
| Fe intake (mg/day) | 8.2 | 2.7 | 11 | 8.3 | 2.5 | 6 |  | 8.1 | 2.2 | 3 | 8.4 | | 2.7 | 5 |
| Vitamin A intake (µg/day) | 1056 | 735.6 | 11 | 1102.2 | 775.3 | 6 |  | 1016.2 | 602.4 | 3 | 1129.4 | | 788.4 | 5 |
| Vitamin D intake (µg/day) | 11.1 | 2.7 | 11 | 11.3 | 2.6 | 6 |  | 11.4 | 2.6 | 3 | 11.4 | | 2.8 | 5 |
| Vitamin K intake (µg/day) | 259.3 | 160.4 | 11 | 277.3 | 178.3 | 6 |  | 236 | 155.7 | 3 | 266.5 | | 181.8 | 5 |
| Vitamin B1 intake (mg/day) | 0.9 | 0.2 | 11 | 0.9 | 0.2 | 6 |  | 0.9 | 0.2 | 3 | 0.9 | | 0.2 | 5 |
| Vitamin B2 intake (mg/day) | 1.2 | 0.5 | 11 | 1.2 | 0.5 | 6 |  | 1.1 | 0.4 | 3 | 1.2 | | 0.5 | 5 |
| Vitamin B6 intake (mg/day) | 1.1 | 0.3 | 11 | 1.1 | 0.3 | 6 |  | 1.1 | 0.3 | 3 | 1.1 | | 0.3 | 5 |
| Vitamin B12 intake (µg/day) | 5.8 | 4.3 | 11 | 5.9 | 4.3 | 6 |  | 5.5 | 3.6 | 3 | 6 | | 4.3 | 5 |
| Folic acid intake (µg/day) | 247.2 | 99.5 | 11 | 255.4 | 105.2 | 6 |  | 239.1 | 86.9 | 3 | 254.9 | | 105.6 | 5 |
| Vitamin C intake (mg/day) | 79.2 | 30.2 | 11 | 80.8 | 27 | 6 |  | 78.9 | 28.2 | 3 | 79.5 | | 26.5 | 5 |
| Mg intake (mg/day) | 204 | 49.5 | 11 | 206.6 | 51.8 | 6 |  | 197.4 | 48.7 | 3 | 201.3 | | 52.1 | 5 |

**Supplementary Table 4. Changes in MMSE scores at weeks 0, 72, and 120 in patients with frailty**

|  | Cognitive Frailty (N=514) | | | Physical Frailty (N=204) | | |
| --- | --- | --- | --- | --- | --- | --- |
|  | Mean | SD | Missing | Mean | SD | Missing |
| 0 weeks | 23.9 | 4.2 | 0 | 24.4 | 5.8 | 0 |
| 72 weeks | 24.8 | 3.7 | 206 | 25.3 | 4 | 80 |
| 120 weeks | 24.4 | 4.4 | 265 | 24.4 | 4.9 | 100 |

**Supplementary Table 5. Frequency of adherence-related treatment discontinuation by treatment group and frailty**

|  | MMSE of 27 or less (N=514) | | | More than 27 (N=455) | | |  |  | Nursing required (N=204) | | | Nursing not required (N=780) | | |  |
| --- | --- | --- | --- | --- | --- | --- | --- | --- | --- | --- | --- | --- | --- | --- | --- |
|  | % | Frequency | N | % | Frequency | N | *p* |  | % | Frequency | N | % | Frequency | N | *p* |
| Teriparatide | 29.1% | 74 | 254 | 29.6% | 66 | 223 | 0.92 |  | 26.6% | 29 | 109 | 30.3% | 115 | 379 | 0.48 |
| Alendronate | 31.5% | 82 | 260 | 20.7% | 48 | 232 | <0.01 |  | 30.5% | 29 | 95 | 25.4% | 102 | 401 | 0.3 |
| Total | 30.4% | 156 | 514 | 25.1% | 114 | 455 | 0.07 |  | 28.4% | 58 | 204 | 27.8% | 217 | 780 | 0.86 |

**Supplementary Table 6. Multivariable logistic regression analysis of associations between adherence-related treatment discontinuation and parameters on univariable analysis**

| Treatment | Predictors | Odds ratio | 95% CI | | *p* |
| --- | --- | --- | --- | --- | --- |
| All | MMSE (1-point increase) | 0.95 | 0.92 | 0.99 | 0.01 |
|  | Count of prevalent vertebral fractures (1-count increase) | 0.91 | 0.84 | 0.99 | 0.03 |
|  | Dyslipidemia | 0.5 | 0.32 | 0.79 | <0.01 |
|  | Weight (1-kg increase) | 0.98 | 0.96 | 1 | 0.03 |
|  | P1NP (1-μg/L increase) | 1.01 | 1 | 1.01 | 0.01 |
|  | TRACP-5b (1-mU/dL increase) | 1 | 1 | 1 | 0.47 |
|  | Triglyceride (1-mg/dL increase) | 1 | 1 | 1 | 0.47 |
|  | eGFR (1-mL/min/1.73m^2^ increase) | 1.01 | 1 | 1.02 | 0.06 |
|  | Albumin (1-g/dL increase) | 0.97 | 0.59 | 1.59 | 0.9 |
|  | Ca (1-mg/dL increase) | 0.72 | 0.49 | 1.06 | 0.1 |
|  | Protein intake | 1.01 | 0.99 | 1.04 | 0.36 |
|  | Fe intake | 0.93 | 0.81 | 1.07 | 0.3 |
|  | Vitamin A intake | 1 | 1 | 1 | 0.84 |
|  | Vitamin B1 intake | 0.46 | 0.01 | 18.9 | 0.68 |
|  | Vitamin B2 intake | 0.71 | 0.24 | 2.07 | 0.52 |
|  | Vitamin B6 intake | 1.29 | 0.19 | 8.97 | 0.8 |
|  | Vitamin B12 intake | 1.03 | 0.96 | 1.11 | 0.36 |
|  | Vitamin B12 intake | 1 | 1 | 1.01 | 0.38 |
|  | Folic acid intake | 0.91 | 0.84 | 0.98 | 0.01 |
|  | Number of teeth extracted last year | 1 | 0.95 | 1.05 | 0.95 |
| Teriparatide | MMSE (1-point increase) | 0.89 | 0.79 | 1.01 | 0.06 |
|  | Count of prevalent vertebral fractures (1-count increase) | 0.53 | 0.29 | 0.99 | 0.05 |
|  | Dyslipidemia | 0.98 | 0.95 | 1.01 | 0.14 |
|  | Weight (1-kg increase) | 1.01 | 1 | 1.01 | 0.07 |
|  | P1NP (1-μg/L increase) | 1 | 1 | 1 | 0.66 |
|  | TRACP-5b (1-mU/dL increase) | 1 | 1 | 1 | 0.82 |
|  | Triglyceride (1-mg/dL increase) | 1.01 | 1 | 1.02 | 0.17 |
|  | eGFR (1-mL/min/1.73 m^2^ increase) | 0.92 | 0.44 | 1.91 | 0.83 |
|  | Albumin (1-g/dL increase) | 0.54 | 0.3 | 0.96 | 0.03 |
|  | Ca (1-mg/dL increase) | 1 | 0.97 | 1.04 | 0.84 |
|  | Protein intake | 0.88 | 0.72 | 1.07 | 0.2 |
|  | Fe intake | 1 | 1 | 1 | 0.93 |
|  | Vitamin A intake | 6.11 | 0.04 | 1035.74 | 0.49 |
|  | Vitamin B1 intake | 1.23 | 0.26 | 5.81 | 0.8 |
|  | Vitamin B2 intake | 0.29 | 0.02 | 4.54 | 0.38 |
|  | Vitamin B6 intake | 1.05 | 0.96 | 1.15 | 0.31 |
|  | Vitamin B12 intake | 1 | 0.99 | 1.01 | 0.67 |
|  | Vitamin B12 intake | 0.91 | 0.82 | 1.02 | 0.1 |
|  | Folic acid intake | 0.9 | 0.85 | 0.95 | <0.01 |
|  | Number of teeth extracted last year | 0.93 | 0.83 | 1.04 | 0.22 |
| Alendronate | MMSE (1-point increase) | 0.38 | 0.19 | 0.77 | 0.01 |
|  | Count of prevalent vertebral fractures (1- count increase) | 0.98 | 0.95 | 1 | 0.09 |
|  | Dyslipidemia | 1.01 | 1 | 1.01 | 0.07 |
|  | Weight (1-kg increase) | 1 | 1 | 1 | 0.51 |
|  | P1NP (1-μg/L increase) | 1 | 0.99 | 1 | 0.44 |
|  | TRACP-5b (1-mU/dL increase) | 1.01 | 0.99 | 1.02 | 0.3 |
|  | Triglyceride (1-mg/dL increase) | 1.09 | 0.53 | 2.22 | 0.82 |
|  | eGFR (1-mL/min/1.73 m^2^ increase) | 0.93 | 0.53 | 1.64 | 0.8 |
|  | Albumin (1-g/dL increase) | 1.02 | 0.99 | 1.06 | 0.21 |
|  | Ca (1-mg/dL increase) | 0.95 | 0.77 | 1.16 | 0.6 |
|  | Protein intake | 1 | 1 | 1 | 0.49 |
|  | Fe intake | 0.03 | 0 | 7.88 | 0.21 |
|  | Vitamin A intake | 0.43 | 0.09 | 2.09 | 0.3 |
|  | Vitamin B1 intake | 4.91 | 0.26 | 91.33 | 0.29 |
|  | Vitamin B2 intake | 1 | 0.89 | 1.12 | 0.96 |
|  | Vitamin B6 intake | 1 | 0.99 | 1.01 | 0.39 |
|  | Vitamin B12 intake | 0.9 | 0.81 | 1.01 | 0.06 |
|  | Vitamin B12 intake | 0.95 | 0.92 | 0.99 | 0.01 |
|  | Folic acid intake | 0.91 | 0.84 | 0.99 | 0.03 |
|  | Number of teeth extracted last year | 0.5 | 0.32 | 0.79 | <0.01 |

*Predictors were selected by univariable logistic regression.
